# Outcome of magnetic resonance guided stereotactic body radiotherapy for hepatocellular carcinoma - an R-IDEAL stage IIb trial

**DOI:** 10.1016/j.ctro.2026.101346

**Published:** 2026-11-02

**Authors:** Marcel Büttner, Laura Uder, Marcel Nachbar, Monica Lo Russo, David Mönnich, Moritz Schneider, Jessica Boldt, Sarah Kübler, Sabrina Baumeister, Michael Bitzer, Silvio Nadalin, Rüdiger Hoffmann, Daniela Thorwarth, Maximilian Niyazi, Cihan Gani, Simon Boeke

**Affiliations:** a Department of Radiation Oncology, University Hospital and Medical Faculty, Eberhard Karls University Tübingen, Tübingen, Germany; b German Cancer Consortium (DKTK), partner site Tübingen; and German Cancer Research Center (DKFZ), Heidelberg, Germany; c Section for Biomedical Physics, Department of Radiation Oncology, University Hospital and Medical Faculty, Eberhard Karls University Tübingen, Tübingen, Germany; d Department of Internal Medicine I, University Hospital Tübingen, Tübingen, Germany; e Department of General, Visceral and Transplant Surgery, University Hospital Tübingen, Tübingen, Germany; f Department of Diagnostic and Interventional Radiology, University of Tübingen, Germany

**Keywords:** hepatocellular carcinoma, Magnetic resonance-guided radiotherapy, Stereotactic body radiotherapy, Online adaptive radiotherapy, Magnetic resonance linear accelerator, Real-time magnetic resonance imaging, Respiratory motion management, Local control, Bridging therapy to liver transplantation, Prospective cohort study

## Abstract

**Introduction:**

Stereotactic body radiation therapy (SBRT) for hepatocellular carcinoma (HCC) is gaining increasing importance as a non-invasive, ablative treatment. However, there are still limited data on online magnetic resonance imaging-guided SBRT (MRgSBRT) for HCCs using MR linear accelerators. The aim of this study was to evaluate the feasibility, safety, and oncological outcomes of online adaptive MRgSBRT in patients with HCC.

**Material and methods:**

All patients who underwent liver MR-guided SBRT for HCC using fraction doses of at least 5 Gy between August 2019 and September 2024 were included. All patients were treated on a 1.5 T magnetic resonance (MR-)-Linac with MR imaging for online adaptation and motion monitoring at each fraction. Treatment-related parameters, local control (LC), overall survival (OS) and progression-free survival (PFS) are reported.

**Results:**

A total of 45 patients with 51 lesions were treated with online adaptive MRgSBRT. Median age was 72 (interquartile range, IQR 67 to 78) years. Liver-directed treatments prior to the SBRT had been applied in 53% of patients. A median of 35 (IQR of 35 to 40) Gray (Gy) was applied with a median of 5 fractions. Median follow-up was 28 months (95% confidence interval, CI: 21–35). LC at 12 and 24 months was 94% (95% CI: 86–100) and 90% (95% CI 79–100). OS after 12 and 24 months were 84% (95% CI: 73–96) and 70% (95% CI: 56–87) and PFS was 54.7% (95% CI: 41–74) and 29% (95% CI: 17%–50%). Of the 14 patients treated for bridging 8 patients (57%) proceeded to receive liver transplantation. No acute adverse events beyond CTCAE grade 2 were documented.

**Conclusion:**

Non-invasive, online adaptive MRgSBRT for HCC in this prospective cohort of patients proved to be well tolerated with excellent local control and is therefore a viable treatment option for patients with HCC.

## Highlights

• MR-guided SBRT achieved 90% local control at 2 years in patients with hepatocellular carcinoma.
• Treatment was well tolerated, with no acute adverse events of CTCAE grade ≥ 3.
• MR guidance facilitated a markerless treatment workflow with real-time motion monitoring.

## 1 Introduction

Online magnetic resonance (MR)-guided adaptive radiotherapy represents a recent technological advance in radiation oncology. MR-Linac hybrid devices provide superior soft-tissue visualization compared with conventional cone-beam CT-based image guidance and enable real-time imaging during treatment delivery. These features are particularly attractive for stereotactic body radiation therapy (SBRT) of liver tumors, where reliable target visualization is challenging and respiratory motion needs to be accounted for. In addition, daily changes in the spatial relationship between the target and adjacent organs at risk, particularly the stomach, duodenum, and bowel, may influence the dose that can safely be delivered. Online MR guidance potentially allows treatment plans to be adapted to the anatomy of the day, thereby maintaining target coverage while limiting dose to adjacent organs at risk and, in selected patients, facilitating dose escalation. However, the magnitude of these potential benefits depends on the anatomical location of the tumor and the adaptive workflow used [1], [2], [3], [4], [5].

Hepatocellular carcinoma (HCC) is often challenging to treat because many patients present with relevant comorbidities and impaired baseline liver function, limiting available therapeutic options. Our group previously demonstrated the feasibility and patient acceptance of online MR-guided radiotherapy for liver metastases [6]. However, clinical data on local control, survival, and adverse events after MR-guided SBRT (MRgSBRT) specifically for HCC remain limited. Therefore, the aim of the present study was to evaluate the feasibility, safety, and oncological outcomes of MRgSBRT in patients with HCC.

## 2 Materials and methods

### 2.1 Patient selection

In this R-IDEAL stage IIb study we included consecutive patients with HCCs who were referred for SBRT after multidisciplinary discussion between August 2019 and September 2024 and treated with fraction doses of 5 Gy or more [7]. Treatments took place within one of two prospective trials on (NCT04172753 (“MRL-01 trial”) or NCT04075305 “MOMENTUM trial”). Both studies were approved by the institutional review board of the Medical Faculty Tübingen (IRB 659/2017BO1 and 059/2023B01). Written informed consent was obtained from all patients.

### 2.2 Treatment planning and radiotherapy workflow

A detailed description of the treatment planning and online adaptive workflow was published previously for liver metastases, and the same workflow was applied in this cohort [6]. In short, treatment simulation was performed with a four-dimensional computed tomography (4D-CT) simulation scan and an MR simulation at the MR-Linac on the same day. Delineation of an internal target volume (ITV), reference plan creation and all online adaptive plans were created with Monaco ® V.5.4, combining all available imaging and clinical data. Individual planning target volume (PTV) margins of three to six millimeters were used depending on the expected positional variability.

The prescription dose was determined based on the ratio of tumor volume to uninvolved liver volume, the absolute uninvolved liver volume, and the predicted dose to the uninvolved liver derived from treatment planning. Dose guidance for organs of interest (OoIs) was primarily based on the UK SABR consensus [8]. Reference plans were created on the exhale phase of the 4D-CT with step and shoot intensity-modulated radiation therapy (IMRT) and five to eleven beams. Various MR-sequences (navigated without and with fat suppression, unnavigated T2-weighted and T1-weighted images) were acquired during simulation at the MR -Linac and fused to the planning CT. In case of poor visibility diagnostic images were fused as well.

In general, online adaptive treatment was based on a free-breathing T2-weighted image and the adapt-to-position (ATP) workflow was performed to account for positional shifts. During treatment delivery a 5 hertz (Hz) cine image with the PTV-contour shown was acquired to ensure target coverage with the possibility of beam interruption in case of insufficient target coverage. The biologically effective dose (BED_10_) was calculated using the linear-quadratic model, assuming an α/β ratio of 10 Gy for tumor tissue.

### 2.3 Statistical aspects

Continuous data were summarized as medians with interquartile ranges (IQR) unless reported otherwise. Adverse events were recorded according to the common terminology of adverse events (CTCAE), version 5.0.

Time-to-event data were calculated from the last radiotherapy treatment according to Kaplan-Meier. Follow-up duration was determined using the inverse Kaplan-Meier method. For local control (LC), progression of the treated lesion was the only event. For progression-free survival (PFS), any progression or death from any cause was considered an event. For overall survival (OS), death from any cause was considered an event. Time-to-event estimates at specific time-points are reported together with 95% confidence intervals (95% CI). Statistics were performed using SPSS, Version 28, IBM, Armonk, New York, figures were created in R, Version 4.4.2. A *p*-value of less than 0.05 was considered statistically significant.

## 3 Results

### 3.1 Patient and treatment characteristics

A total of 45 consecutive patients meeting aforementioned criteria were treated with online adaptive MR-guided radiotherapy for HCC between August 2019 and September 2024.

Patient characteristics are summarized in Table 1. Median patient age was 72 years (IQR 67–78 years) and most patients presented with Child-Pugh class A cirrhosis (33/45, 73%). In total 24 patients (53%) had received liver-directed local treatment prior to the SBRT. In 39 patients (87%) a single lesion was treated, six patients (13%) received treatment of more than one liver lesion using separate treatment plans. In 13 patients the target volume was in close proximity of luminal organs (bowel or stomach). Most patients (40/45, 89%) were treated in five fractions, while three patients (7%) received three fractions, one patient (2%) received four fractions, and one patient (2%) received six fractions. The median prescribed dose was 35 Gy (IQR, 35–40 Gy). The most common fractionation schedules were 35 Gy in 5 fractions (*n* = 17), 50 Gy in 5 fractions (*n* = 10), and 40 Gy in 5 fractions (*n* = 7). The complete distribution of dose and fractionation schedules is provided in Table 1. Prescription dose and fractionation were individualized according to tumor size, uninvolved liver volume and function, and predicted dose to the uninvolved liver and adjacent organs of interest.

**Table 1:** Patient characteristics, tumor-related parameters and treatment regimens.⁎

Patient characteristics, tumor-related parameters and treatment regimens	
	n
Number of patients	45
Number of treated lesions	51
	n (%)
Sex	
Female	7 (16%)
Male	38 (84%)
Median age at initial diagnosis (IQR)	72 (67–78)
Number of treated lesions	
*n* = 1	39 (87%)
*n* > 1	6 (13%)
Distant (extrahepatic) metastases	
Yes	3 (7%)
No	42 (93%)
Child-Pugh score	
Child-Pugh A	33 (73%)
Child-Pugh B	9 (20%)
Child-Pugh C	3 (7%)
SBRT for bridging	
Yes	14 (31.1%)
No	31 (68.9%)
Systemic treatment prior to RT	
No	40 (89%)
Sorafenib	4 (9%)
Atezolizumab	1 (2%)
Previous liver-directed therapy	
No	21 (47%)
Yes (Surgery, TACE, RFA, Radiotherapy*)	24 (53%)
Number of fractions	
5 fractions	40 (88.9%)
3 fractions	3 (6.7%)
4 fractions	1 (2.2%)
6 fractions	1 (2.2%)
Dose and fractionation schedule	
35 Gy in 5 fractions	17 (37.8%)
50 Gy in 5 fractions	10 (22.2%)
40 Gy in 5 fractions	7 (15.6%)
37.5 Gy in 3 fractions	3 (6.7%)
30 Gy in 5 fractions	3 (6.7%)
25 Gy in 5 fractions	3 (6.7%)
30 Gy in 4 fractions	1 (2.2%)
30 Gy in 6 fractions	1 (2.2%)

IQR-Interquartile range, TACE-Transcatheter arterial chemoembolization, RFA-Radiofrequency ablation.

⁎ Palliative radiotherapy for superior vena cava syndrome

### 3.2 Oncological outcomes

Volumetric and dosimetric parameters are summarized in Table 2. The median applied BED_10_ GTV D98 was 67.6 Gy (IQR 61.9–102.0) and median applied BED_10_ ITV D98 was 66.3 Gy (57.8–100.6). All doses were prescribed according to institutional protocol to the target volume, typically covering the planning target volume at the 80% isodose line (IDL) or following ICRU specifications. No acute CTCAE grade > 2 adverse events were observed. Median follow-up was 28 months (95% CI: 21–35). LC at 12 and 24 months was 94% (95% CI: 86–100) and 90% (95% CI 79–100), Fig. 1a. OS after 12 and 24 months was 84% (95% CI: 73–96) and 70% (95% CI: 56–87) and PFS was 54.7% (95% CI: 41–74) and 29% (95% CI: 17%–50%), Fig. 1b and c. Twenty-four patients (53%) had intrahepatic progression outside of the treated lesion. Of the patients who had out-of-field distant intrahepatic recurrence, three patients received repeat SBRT for the recurrent lesion. Of the 14 patients who received SBRT as a bridging procedure, eight (57%) underwent transplantation.

**Table 2:** Dose-volume histogram parameters for target volumes and normal tissues. Values are reported as median, maximum, and interquartile range (IQR). BED₁₀ denotes the biologically effective dose calculated assuming an α/β ratio of 10 Gy. GTV, gross tumor volume; ITV, internal target volume; PTV, planning target volume; D98, minimum dose covering 98% of the target volume; V10, percentage volume receiving at least 10 Gy.

Structure	Parameter	Median	Max	IQR
GTV	Volume [cm^3^]	16,4	50,2	4,7 - 27,8
	Mean dose [Gy]	40,0	60,5	37,9 - 51,7
	Max dose [Gy]	42,4	64,3	40,1 - 53,9
	D98 [Gy]	38,3	59,3	35,8 - 47,5
	BED D98 [Gy]	67,6	129,5	61,9 - 102,0
ITV	Volume [cm^3^]	22,1	86,7	8,9 - 47,3
	D98 [Gy]	37,8	59	34,3 - 47,1
	BED D98 [Gy]	66,3	128,6	57,8 - 100,6
PTV	Volume [cm^3^]	42,9	124,3	28,3 - 80,6
	D98 [Gy]	34,0	51,2	30,9 - 38,1
	BED D98 [Gy]	56,1	93,5	47,8 - 59,4
Uninvolved liver (Liver-GTV/ITV)	Volume [cm^3^]	1424,9	2580,6	1225,3 - 1809,6
	Mean dose [Gy]	7,2	12,8	5,0 - 9,0
	V10 [%]	24,0	54,3	15,9 - 36,8
Bowel	D0.5 cm^3^ [Gy]	12,0	34,7	4,6 - 26,4
	D0.035 cm^3^ [Gy]	13,3	36,3	5,8 - 30,4

**Fig. 1:**
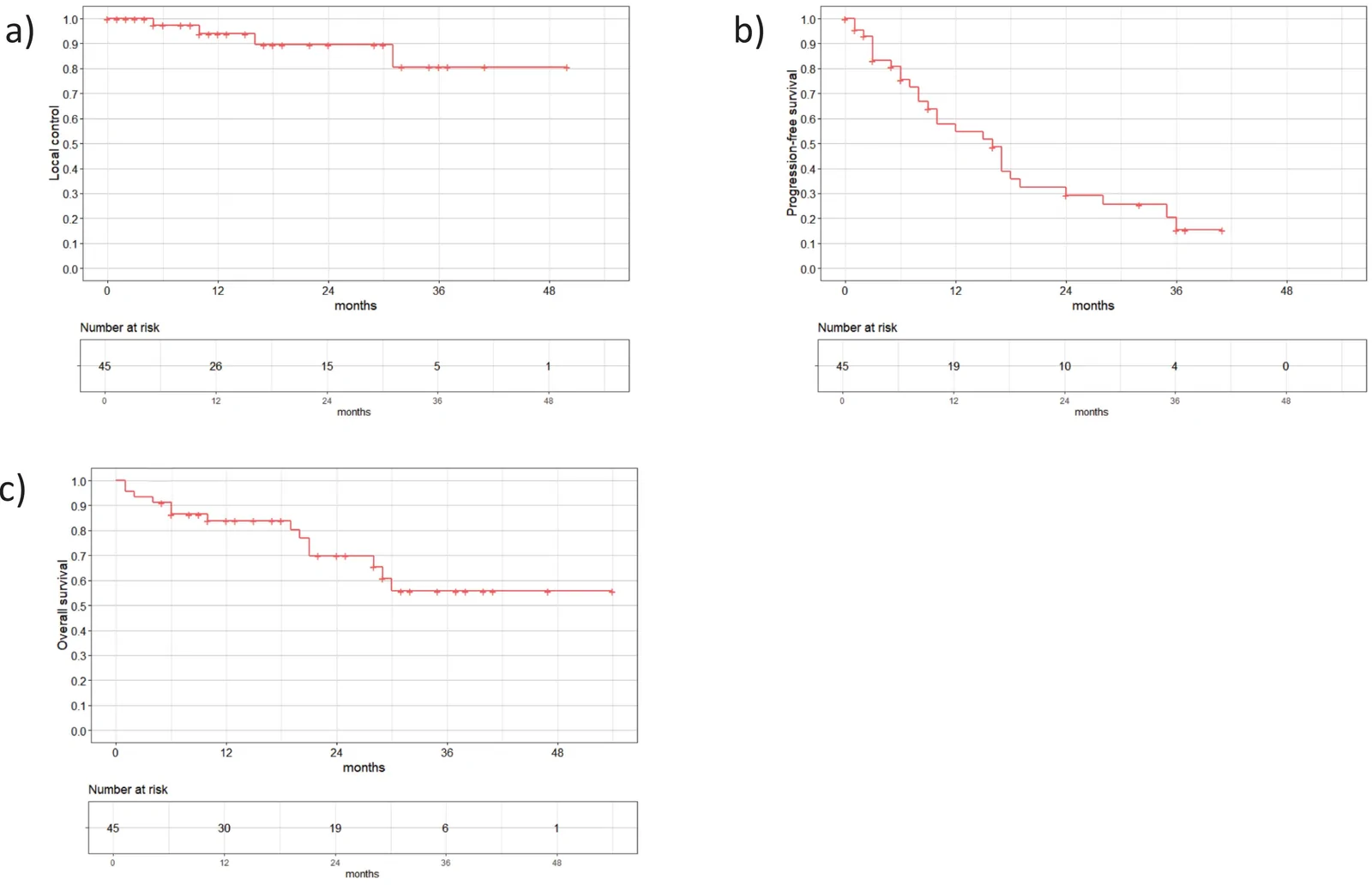
a) Local control of the irradiated liver lesion (LC), b) Progression-free survival (PFS), c) Overall survival (OS).

## 4 Discussion

In the present study, we report oncological outcomes in patients with HCC treated with MRgSBRT. In accordance with our institutional practice and national guidelines, patients were generally referred for SBRT when surgery, radiofrequency ablation (RFA), or transarterial chemoembolization (TACE) was considered unsuitable or associated with a high risk of local failure. Despite this selection of patients with frequently unfavorable clinical or tumor characteristics, local control was excellent, reaching 94% at one year and 90% at two years.

Clinical evidence specifically addressing MR-guided SBRT for HCC remains limited, as most published series of MR-guided liver radiotherapy have included heterogeneous cohorts of primary and metastatic liver tumors. Weykamp et al., for example, reported a one-year local control rate of 88.1% in 20 patients undergoing MR-guided SBRT, of whom two had HCC [9]. In another series, six of 26 patients treated for liver malignancies had HCC, with an overall one-year local control rate of 100% [10]. Beyond MR-guided radiotherapy, the evidence supporting SBRT for HCC continues to grow. Xi et al. recently demonstrated superior local progression-free survival with SBRT compared with RFA in patients with solitary recurrent HCC ≤5 cm, while the TRENDY trial demonstrated superior local control with SBRT compared with TACE [11], [12]. These findings are also supported by a recent systematic review and meta-analysis [13].

Fourteen patients in our study received SBRT as a bridge to transplant. SBRT and TACE share the advantage over RFA that no invasive manipulation of the tumor occurs potentially leading to tumor spread [14]. At the time of this report, eight patients have successfully undergone transplantation. This finding is in line with the report from Wong and colleagues who treated 40 patients with SBRT as bridging therapy within a prospective study [15]. Of those 40 patients 27 were successfully transplanted. Dropouts from listing were significantly less frequent after SBRT compared with a historical control of patients who received TACE for transplant. Moreover, no difference in 30-day mortality or adverse events after SBRT or TACE was observed. Similarly, Sapisochin et al. reported an excellent transplant rate (30 of 36 patients transplanted) and a low drop-out rate of 16.7% for patients who had SBRT as a bridge to transplant [16].

Most patients in our cohort received a BED₁₀ below 100 Gy because of limitations related to uninvolved liver volume, liver function, or adjacent organs at risk. Evidence regarding a dose-response relationship for SBRT in HCC remains conflicting. In an international individual-patient-data meta-analysis, Ohri et al. observed improved local control with BED₁₀ ≥100 Gy for liver metastases but not for primary liver tumors [17]. In contrast, Su et al. recommended a BED₁₀ of at least 100 Gy based on a pooled analysis of 602 patients with HCC treated in China [18].

Differences in patient populations and underlying liver disease may partly contribute to these discrepant observations. The etiology of HCC varies considerably across geographic regions, with chronic hepatitis B virus infection accounting for a larger proportion of cases in Eastern Asia, whereas hepatitis C virus infection, alcohol-related liver disease, and metabolic liver disease have historically contributed more prominently in Western populations [19]. These differences are associated with variations in the prevalence and severity of cirrhosis, liver function, tumor characteristics, and competing risks, all of which may influence patient selection, radiation tolerance, and outcomes after SBRT. Consequently, validation of dose-response relationships across different patient populations remains important.

This study has several limitations. All patients were treated using the Adapt-to-Position (ATP) workflow, which relies on a virtual couch shift, rather than the Adapt-to-Shape (ATS) workflow with re-segmentation and plan adaptation at each treatment fraction. This approach was chosen because of the observed anatomical stability and the limited changes in tumor volume during treatment. The ATP workflow was therefore favored to avoid prolongation of treatment times associated with repeated recontouring and online replanning. In addition, low-grade adverse events were not systematically recorded. Patient-reported outcome measures were collected but will be reported separately. Furthermore, the single-center design and the absence of a comparator group limit conclusions regarding the relative effectiveness of MR-guided SBRT compared with conventional SBRT or other local treatment modalities.

One limitation of online MR-guided radiotherapy is the need to acquire daily MR imaging and perform online plan adaptation, which may prolong treatment times. An additional challenge is respiratory motion of the liver, which can either be addressed by ITV-based approaches or gating procedures. However, our study and previous reports on both high-field and low-field MR-guided radiotherapy have consistently shown high patient acceptance and feasibility despite treatment within an MRI [6], [9], [20].

In summary, we were able to show that online MR-guided SBRT is a well-tolerated and highly effective fully non-invasive local treatment with a favorable safety profile both for definitive treatment and as a bridge to transplant.

## Declaration of generative AI and AI-assisted technologies in the manuscript preparation process

During the preparation of this work, the author(s) used chatGPT for language editing and coding. The author(s) reviewed and edited the output as needed and take full responsibility for the content of the published article.

## Ethics declaration

Written informed consent to take part in the study and to publish the article has been obtained from all participants or their legal representatives. The privacy rights of participants have been observed.

This study was performed in compliance with relevant laws, regulatory frameworks and guidelines where the research took place.

This study was approved by the IRB of the University of Tübingen.

(Approval No. IRB 659/2017BO1 and 059/2023B01)

This clinical trial was registered with number NCT04075305 (clinicaltrials).

## Declaration of competing interest

The Department of Radiation Oncology Tübingen receives within the frame of research agreements financial and technical support as well as sponsoring for travels and scientific symposia from Elekta AB (Stockholm, Sweden). CG declares honoraria and travel support from Elekta AB.
